# Dissecting the biology of gliomagenesis: Evaluating the interaction between *IDH* tumor mutation and germline variants

**DOI:** 10.1093/noajnl/vdaf147

**Published:** 2025-07-08

**Authors:** Matthew L Kosel, Paul A Decker, Thomas M Kollmeyer, Kristen L Drucker, Anne K Shurtz, Annette M Molinaro, Gian Marco Conte, Mana Moassefi, Bradley J Erickson, John K Wiencke, Stephen Francis, Terry C Burns, Rachel A Vaubel, Margaret Wrensch, Daniel H Lachance, W Oliver Tobin, Robert B Jenkins, Jeanette E Eckel-Passow

**Affiliations:** Department of Quantitative Health Sciences, Mayo Clinic, Rochester, MN, USA; Department of Quantitative Health Sciences, Mayo Clinic, Rochester, MN, USA; Department of Laboratory Medicine and Pathology, Mayo Clinic, Rochester, MN, USA; Department of Laboratory Medicine and Pathology, Mayo Clinic, Rochester, MN, USA; Department of Quantitative Health Sciences, Mayo Clinic, Rochester, MN, USA; Department of Epidemiology and Biostatistics, University of California, San Francisco, San Francisco, CA, USA; Department of Neurological Surgery, University of California, San Francisco, San Francisco, CA, USA; Department of Radiology, Mayo Clinic, Rochester, MN, USA; Department of Radiology, Mayo Clinic, Rochester, MN, USA; Department of Radiology, Mayo Clinic, Rochester, MN, USA; Department of Epidemiology and Biostatistics, University of California, San Francisco, San Francisco, CA, USA; Department of Neurological Surgery, University of California, San Francisco, San Francisco, CA, USA; Department of Epidemiology and Biostatistics, University of California, San Francisco, San Francisco, CA, USA; Department of Neurological Surgery, University of California, San Francisco, San Francisco, CA, USA; Department of Neurologic Surgery, Mayo Clinic, Rochester, MN, USA; Department of Laboratory Medicine and Pathology, Mayo Clinic, Rochester, MN, USA; Department of Neurological Surgery, University of California, San Francisco, San Francisco, CA, USA; Department of Neurology, Mayo Clinic, Rochester, MN, USA; Department of Laboratory Medicine and Pathology, Mayo Clinic, Rochester, MN, USA; Mayo Clinic Center for Multiple Sclerosis and Autoimmune Neurology, Mayo Clinic, Rochester, MN, USA; Department of Neurology, Mayo Clinic, Rochester, MN, USA; Department of Laboratory Medicine and Pathology, Mayo Clinic, Rochester, MN, USA; Department of Quantitative Health Sciences, Mayo Clinic, Rochester, MN, USA

**Keywords:** GWAS, polygenic risk score, *ROBO*, *PHLDB1*, *D2HGDH*

## Abstract

**Background:**

The *CCDC26* germline variant rs55705857 is causal for development of *IDH* mutant (*IDH*mut) adult glioma. However, ~60% of *IDH*mut patients do not carry the rs55705857 risk allele. We aimed to identify variants associated with developing *IDH*mut glioma among patients that do not have the rs55705857 risk allele and to further understand development of *IDH*wt glioma.

**Methods:**

We used three datasets that included 1216 *IDH*mut and 1442 *IDH*wt glioma patients and a case–case design to perform genome-wide association (GWAS) analyses comparing *IDH*mut versus *IDH*wt glioma. Analyses were performed overall and stratified by rs55705857 genotype and sex. Multivariable logistic regression and regression trees were used to develop models to predict *IDH* status using germline variants, age, and contrast enhancement on MRI.

**Results:**

Three regions were identified comparing *IDH*mut versus *IDH*wt: rs55705857 (meta *P*-value [*P*] = 1.35 × 10^−43^), *PHLDB1* (rs7125115, *P* = 3.46 × 10^−17^), and *D2HGDH* (rs71430382, *P* = 2.43 × 10^−12^). When analyzing only patients that do not have the rs55705857 risk allele, *PHLDB1* (rs7125115, *P* = 1.73 × 10^−13^) and *D2HGDH* (rs71430382, *P* = 8.86 × 10^−10^) were identified. Among patients who have the rs55705857 risk allele, four variants between *ROBO1* and *ROBO2* (rs4680975, *P* = 4.65 × 10^−7^) increased the likelihood of having an *IDH*wt tumor. Tumor expression of *ROBO1* was associated with rs4680975 genotype in *IDH*wt patients that have the rs55705857 risk allele (*P* = 0.034). Multivariable logistic analysis demonstrated that rs55705857, rs71430382 (*D2HGDH*), and age predicted *IDH* mutation status.

**Conclusions:**

To understand the development of adult glioma, we demonstrate that *D2HGDH* and *PHLDB1* should be prioritized for functional studies in *IDH*mut tumors. The *ROBO1* region warrants further investigation in *IDH*wt tumors.

**Key Points**

- *D2HGDH* and *PHLDB1* regions should be prioritized for functional experiments for *IDH*mut tumors.
- *ROBO* variant rs4680975 should be evaluated for development of *IDH*wt tumors.
- Germline variants and tumor acquired alterations are ancestry specific.

**Importance of the Study**

This study identified interactions between tumor *IDH* mutation and germline variants in or near *CCDC26*, *D2HGDH*, *PHLDB1,* and *ROBO1/2*. The *ROBO1/2* variants are novel and were not previously identified when a case–control GWAS was performed stratified by *IDH* tumor status; thus, demonstrating the importance of performing a more efficient case–case analysis to evaluate interactions. A polygenic risk model was developed to predict *IDH* tumor mutation status. The model contained two germline variants and age at diagnosis and produced similar accuracy as a prior model that utilized millions of germline variants. Overall, the study highlights three regions that require further functional analyses to determine the causal relationship of *IDH*mut and *IDH*wt glioma and to develop relevant models to aid in therapeutic evaluation.

Understanding how brain tumors develop and how germline alterations predispose to the evolution of specific tumor alterations is important for dissecting the biology of gliomagenesis. Germline variants provide critical information to develop accurate tumor models that can then be used to evaluate novel therapeutics, stratify clinical trials, and develop polygenic models to predict risk of disease and risk of specific tumor molecular alterations. Brain tumor therapeutics for *IDH* wildtype (*IDH*wt) glioblastoma (GBM) have not changed since 2005. With respect to *IDH* mutant (*IDH*mut) glioma, the efficacy of *IDH* inhibitor drugs is being evaluated. Thus, there is a clear clinical need to further understand development of brain tumor subtypes to help move the field forward. The *CCDC26* germline variant rs55705857 has a large odds ratio (OR > 5) for the development of *IDH*mut glioma, is a causal variant for *IDH*mut glioma, and a preclinical mouse model incorporating this variant was developed that can be used to assess novel therapeutics.^1^ Causality of rs55705857 was concluded based on fine mapping and functional mouse experiments, that is, germline variants are classified as causal if they have large effect sizes (high penetrance) and have functional and mechanistic support.^2,3^ However, ~60% of *IDH*mut patients do not carry the rs55705857 risk allele.^1^ This highlights the importance of identifying germline variants that are responsible for the development of *IDH*mut glioma among patients that do not carry the rs55705857 risk allele, as well as identifying variants that are associated with the development of *IDH*wt tumors. Furthermore, the risk allele for rs55705857 is not observed in East Asians^4^ and is observed with very low frequency in other non-European ancestries, demonstrating that there are additional important germline variants associated with development of *IDH*mut tumors (**Table 1**).

**Table 1.** Published Glioma Risk Variants and Allele Frequencies from 1000 Genomes for European, East Asian, South Asian, and African Populations.

1000 Genome Frequency									
Cytoband	Nearby Gene	Variant	Allele	European (*n* = 1006)	East Asian (*n* = 1008)	South Asian (*n* = 978)	African (*n* = 1322)	Case-Control GWAS Population	Glioma Subtype Associations
1p31.3	*RAVER2*	rs12752552	C	0.1302	0.0000	0.020	0.1664	European	*IDH*wt
1q32.1	*MDM4*	rs4252707	A	0.2197	0.4018	0.249	0.1241	European	*IDH*mut
1q44	*AKT3*	rs12076373	C	0.1630	0.3165	0.223	0.3056	European	*IDH*mut
2q33.3	*IDH1*	rs7572263	G	0.2435	0.0278	0.302	0.3828	European	*IDH*mut
2q37.3	*D2HGDH*	rs71430382	T	0.3857	0.4127	0.285	0.6853	European	*IDH*mut
3p14.1	*LRIG1*	rs11706832	C	0.4563	0.1915	0.373	0.0877	European	*IDH*mut
5p15.33	*TERT*	rs10069690	T	0.2763	0.1687	0.271	0.6619	European; East Asian^*^	*IDH*wt; *IDH*mut non-codel
7p11.2	*EGFR*	rs723527	G	0.4274	0.9732	0.649	0.7383	European	*IDH*wt
7p11.2	*EGFR*	rs75061358	G	0.0994	0.0010	0.004	0.0045	European	*IDH*; *IDH*mut 1p/19q codel
7p22.3	*FAM20C*	rs111976262	A	0.0368	0.0099	0.013	0.2769	European	*IDH*mut 1p/19q codel
8q24.21	*CCDC26*	rs55705857	G	0.0567	0.0000	0.022	0.0008	European	*IDH*mut
9p21.3	*CDKN2A/B*	rs634537	G	0.4105	0.0923	0.274	0.0166	European	*IDH*wt
10q24.33	*STN1*	rs11598018	C	0.4622	0.6587	0.593	0.7148	European	*IDH*mut
10q25.2	*VTI1A*	rs11599775	A	0.3797	0.1915	0.175	0.0401	European	*IDH*mut
11q14.1	*FAM181B*	rs11233250	T	0.1322	0.1865	0.263	0.0325	European	*IDH*wt
11q21	*MAML2*	rs7107785	T	0.4791	0.1974	0.219	0.3510	European	*IDH*mut
11q23.2	*ZBTB16*	rs648044	A	0.3897	0.4563	0.482	0.1384	European	*IDH*mut
11q23.3	*PHLDB1*	rs12803321	C	0.3569	0.3175	0.413	0.6195	European; East Asian^*^	*IDH*mut non-codel
12p11.23	*STK38L*	rs10842893	T	0.0775	0.0347	0.046	0.0038	East Asian	All glioma
12q21.2	*PHLDA1*	rs1275600	A	0.4046	0.7827	0.584	0.6362	European	*IDH*mut
14q12	*AKAP6*	rs10131032	A	0.0845	0.1875	0.173	0.0809	European	*IDH*mut
15q21.3	*RAB27A*	rs4774756	C	0.6153	0.2966	0.530	0.8071	East Asian	All glioma
15q24.2	*ETFA*	rs77633900	C	0.0865	0.0575	0.078	0.0023	European	*IDH*mut
16p13.3	*LMF1*	rs3751667	T	0.2078	0.4127	0.446	0.4274	European	*IDH*mut
16p13.3	*MPG/RHBDF1*	rs2562152	A	0.1501	0.3631	0.322	0.7859	European	*IDH*wt
16q12.1	*HEATR3*	rs10852606	T	0.2873	0.3571	0.278	0.4561	European	*IDH*wt
17p13.1	*TP53*	rs78378222	G	0.0129	0.0000	0.000	0.0000	European	All glioma
19p13.12	*CYP4F12*	rs688755	C	0.2763	0.0139	0.222	0.4024	East Asian	All glioma
20q13.33	*RTEL1*	rs2297440	T	0.2038	0.7113	0.266	0.0257	European; East Asian^*^	*IDH*wt
22q13.1	*SLC16A8*	rs2235573	A	0.4930	0.4831	0.343	0.4145	European	*IDH*wt

^*^Validation of European variants in East Asians. The East Asian targeted validation identified a different variant in the region than the variant observed to be the most significant in Europeans.

To further understand the interaction between *IDH* tumor mutation and germline genetics, we performed a case–case GWAS in three independent datasets consisting of 2,658 adult glioma patients and directly compared *IDH*mut versus *IDH*wt patients. A case–case analysis is a statistically efficient method to evaluate interactions between germline variants and *IDH* tumor mutation and does not require normal controls.^5^ We stratified the analysis by rs55705857 genotype to (i) identify germline variants that are associated with development of an *IDH*mut tumor among the ~60% of patients that do not carry the rs55705857 risk allele and (ii) identify variants that are associated with development of an *IDH*wt tumor, including in patients that carry the rs55705857 risk allele. Germline variants, including rs55705857, have been reported to interact with sex.^6,7^ Thus, we also performed analyses stratified by sex.

Germline variants and polygenic risk models have been developed to predict *IDH* tumor mutation status.^8,9^ If a lesion is likely a glioma, then an *IDH* mutation polygenic model would allow for prediction of molecular subtype prior to surgery using a noninvasive blood-based assay. Prior polygenic models were developed using multivariable logistic regression that only considered germline variants that passed a prespecified statistical significance level,^8,9^ or a Bayesian shrinkage model that was agnostic and considered all genomewide variants.^9^ None of the models considered candidate interactions between germline variants, or interactions between a covariate (such as age or sex) and germline variants. We evaluated regression trees for developing a polygenic model to predict *IDH* mutation status, which is a modeling technique that accounts for interactions. In addition to age, we also considered contrast enhancement on MRI as a candidate predictor variable.

## Methods

### Mayo GWAS: *Affymetrix Axiom™ Precision Medicine Diversity Array Plus*

The Mayo Affymetrix GWAS data consisted of 349 *IDH*mut and 351 *IDH*wt adults with diffuse glioma from Mayo Clinic who were genotyped on a custom Affymetrix GWAS genotyping array: Axiom™ Precision Medicine Diversity Array Plus. The Affymetrix array included custom content on 49,955 variants, including all published glioma variants, common variants (minor allele frequency [MAF] >= 5%) within 200 kb from the published glioma variants, and 3,465 variants associated with non-brain tumors.

### Mayo/UCSF GWAS: *Illumina OncoArray*

The Mayo/UCSF Illumina GWAS data consisted of previously described Illumina OncoArray genotyping of 485 *IDH*mut and 686 *IDH*wt adults with diffuse glioma from Mayo Clinic and UCSF.^10^ All Mayo Clinic patients that were included in the Mayo Affymetrix GWAS data were removed from the Mayo/UCSF Illumina GWAS data.

### The Cancer Genome Atlas (TCGA) GWAS: Affymetrix 6.0

TCGA Affymetrix 6.0 data consisted of previously described Affymetrix data from 382 *IDH*mut and 405 *IDH*wt adults.^10^

### Statistical Methods

A case–case GWAS was performed for each GWAS dataset separately comparing *IDH*mut versus *IDH*wt glioma. A case–case GWAS is an efficient method to evaluate interactions between germline variants and an acquired tumor alteration; it results in smaller standard errors and thus requires smaller sample sizes than models that include formal interaction terms.^5^ The odds ratio (OR) from the case–case analysis denotes the ratio of the relative risk for developing an *IDH*mut glioma versus the relative risk for developing an *IDH*wt glioma.^11^ Thus, an OR > 1 denotes that risk is higher in *IDH*mut glioma (vs *IDH*wt glioma) for the specific allele being modeled.

The following GWAS quality-control procedures were applied to each GWAS dataset: tests of Hardy-Weinberg equilibrium (*P*-value [*P*] < 10^-6^), duplicate and relatedness, sex, variant call rates (> 95%), and subject call rates (> 95%). Imputation was performed using the Michigan Imputation Server, utilizing TOPMed. Admixture was applied to determine racial groups, using 1000 Genome samples to anchor the racial groups. Population stratification was assessed using Eigenstrat and principal components. Quality control, imputation, and population stratification was previously applied to the Mayo/UCSF and TCGA data.^10^ Logistic regression was utilized to compare *IDH*mut versus *IDH*wt, among subjects with a probability of European ancestry ≥ 0.75, with genotype coded as 0, 1, or 2 copies (or dosage if imputed) of the modeled allele. For the Mayo Affymetrix GWAS, sex and the first two principal components were included as covariates. For the Mayo/UCSF Illumina GWAS, sex, site (Mayo vs UCSF), and the first two principal components were included as covariates. For the TCGA GWAS, sex, and the first four principal components were included as covariates. Variants that passed a *P*-value threshold of *P* < 1 × 10^-4^ in any of the three GWAS datasets were subsequently meta analyzed. Variants that had the same direction of effect across all three datasets and genomewide significant meta *P*-value (*P* < 5 × 10^−8^) were considered genomewide significant. Variants that had a meta *P* < 5 × 10^−6^ were determined to have nominal level of significance. GWAS were also performed stratified by the *CCDC26* variant rs55705857. Because the primary analysis consisted of a meta-analysis across the three GWAS, an imputation quality threshold was not imposed; however, imputation quality was assessed when reviewing the meta-analysis results. GWAS were performed stratified by sex and a z-statistic was used to test if ORs were different across sex. *LDlink* (https://ldlink.nih.gov) was used to obtain estimates of linkage disequilibrium (LD) across pairs of variants. Multinomial logistic regression was used to perform case–case analyses comparing *IDH*mut 1p/19q-codeleted glioma versus *IDH*wt, as well as *IDH*mut non-codeleted versus *IDH*wt.

Expression quantitative trait loci (eQTL) analyses were performed using data obtained from the GTEx Portal on 11/4/2024. Hi-C analyses were performed using the 3D-genome interaction viewer and database. Interactions were examined for a 2Mb window surrounding the variant of interest in the hippocampus and dorsolateral prefrontal cortex.^12^ ChromHMM was evaluated for the same region in the dorsolateral prefrontal cortex and hippocampus using the UC Santa Cruz Genome Browser and the Roadmap Epigenomics Project.^13^ TCGA RNA expression data were also used to perform eQTL analyses. LGG and GBM expression data from TCGA were analyzed separately since LGG were analyzed using RNA sequencing technology whereas GBM were analyzed using U133 Affymetrix microarrays. Analyses were performed within subtypes of glioma, as defined by *IDH* mutation status and the *CCDC26* variant rs55705857. A *P*-value threshold of .05 was determined to be statistically significant for the targeted eQTL analyses.

To develop polygenic risk models for predicting tumor *IDH* mutation status, recursive partitioning and regression trees (*rpart*; https://github.com/bethatkinson/rpart) were used. Classification and regression trees use simple decision trees to make predictions, and thus by definition, models interactions between variables. The regression trees were developed using Mayo Affymetrix data and validated on the Mayo/UCSF Illumina and TCGA data. Trees were pruned using the minimal complexity parameter that minimized the 10-fold cross validation error. Pruning is a regularization method that tries to avoid overfitting by reducing model complexity. As an additional step to overcome overfitting, only 31 germline variants were considered in developing the regression trees: 27 variants reported in European GWAS, three variants reported in East-Asian GWAS, and the most significant variant near *ROBO1* that was observed in this study to be associated with risk of developing *IDH*wt tumors among the subset of patients who carry at least one rs55705857 risk allele. The interaction between rs55705857 and the *ROBO1* variant demonstrates why it is important to at least consider models that account for interactions. In parallel, multivariable logistic regression with elastic net regularization was also used to develop polygenic risk models. We also evaluated whether age and contrast enhancement on MRI provided improved predictive performance. Area under the receiver operator characteristic curve (AUC), sensitivity, and specificity were used to evaluate predictive performance, where sensitivity denotes the ability to correctly identify patients that are *IDH*mut and specificity denotes the ability to correctly identify patients that are *IDH*wt.

### Population Allele Frequencies of All Known Adult Diffuse Glioma Germline Variants

Population allele frequencies for all published adult diffuse glioma germline variants were obtained from 1000 Genomes for European, East Asian, South Asian, and Africans (release version: 20231103111315). Adult diffuse glioma GWAS have only been performed in European and East Asian populations to date and 30 germline variants have been identified: 27 in European and three in East Asian populations (**Table 1**). Three of the 27 regions identified in Europeans have been validated in an East Asian population; however, a different variant in each of the three regions was observed in East Asians.^14^**Table 1** provides allele frequencies for European, East Asian, South Asian, and African populations for each of the 30 glioma variants and shows that the allele frequencies for most of the variants differ across populations. The two variants with the largest odds ratios in Europeans, *CCDC26* and *TP53*, are rare or not observed at all in the other populations. There are many genes that are known to be altered in brain tumors that also have germline variant associations (eg, *IDH1*, *EGFR*, *CDKN2B*, and *PHLDB1*); the allele frequency of these germline variants also differs across populations. Due to the large variability in allele frequencies for known glioma risk variants across populations, all GWAS analyses in this study were performed within patients of European ancestry as there were too few patients of other genetic ancestries in the three datasets to analyze them on their own.

## Results

### GWAS Case Cohorts

**Figure 1.**
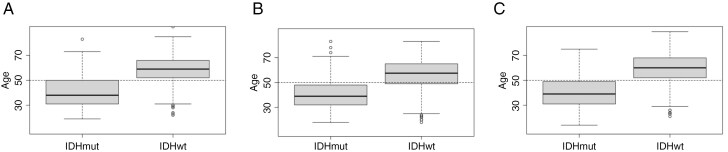
Distribution of age at diagnosis for *IDH*mut and *IDH*wt patients in the (a) Mayo Affymetrix cohort, (b) Mayo/UCSF Illumina cohort, and (c) TCGA. The horizontal reference line represents age of 50 years.

Genotypes for a total of 1,216 *IDH*mut and 1,442 *IDH*wt adults with glioma were analyzed across the three GWAS datasets: (i) Mayo Affymetrix, (ii) Mayo/UCSF Illumina, and (iii) TCGA (**Table 2**). Overall, females represented 43% and 39% of the adults with *IDH*mut and *IDH*wt glioma, respectively. The age at diagnosis was significantly older in *IDH*wt compared to *IDH*mut patients across all three datasets (*P* < .0001; **Figure 1**). Of the 1,130 *IDH*mut glioma patients that had 1p/19q codeletion status available, 473 (42%) were 1p/19q codeleted.

**Table 2.** Demographic Information for the Three GWAS Datasets.

	Mayo Affymetrix		Mayo/UCSF Illumina		TCGA	
	*IDH*mut (*N* = 349)	*IDH*wt (*N* = 351)	*IDH*mut (*N* = 485)	*IDH*wt (*N* = 686)	*IDH*mut (*N* = 382)	*IDH*wt (*N* = 405)
**Sex**						
Female	158 (45.3%)	140 (39.9%)	195 (40.2%)	266 (38.8%)	170 (44.5%)	161 (39.8%)
Male	191 (54.7%)	211 (60.1%)	290 (59.8%)	420 (61.2%)	212 (55.5%)	244 (60.2%)
						
**Age at diagnosis**						
Mean (SD)	40.3 (12.23)	58.4 (11.66)	40.7 (11.76)	56.3 (11.99)	40.8 (12.37)	59.7 (12.74)
Median (IQR)	38.0 (31.0, 50.0)	59.0 (52.0, 66.0)	39.0 (32.0, 48.0)	57.5 (49.0, 65.0)	39.0 (31.0, 49.0)	60.0 (52.0, 68.0)
Range	19.0, 83.0	22.0, 93.0	18.0, 83.0	18.0, 83.0	18.0, 75.0	21.0, 89.0
						
**1p/19q codeletion**						
Codeleted	145 (46.8%)	0 (0.0%)	185 (42.2%)	0 (0.0%)	143 (37.4%)	0 (0.0%)
Not codeleted	165 (53.2%)	167 (100.0%)	253 (57.8%)	505 (100.0%)	239 (62.6%)	397 (100.0%)
Missing	39	184	47	181	0	8
						
**Tumor Grade**, *n* (%)						
2	182 (52.1%)	14 (4.0%)	274 (56.5%)	52 (7.6%)	168 (48.3%)	11 (2.8%)
3	140 (40.1%)	56 (16.0%)	162 (33.4%)	105 (15.3%)	155 (44.5%)	56 (14.1%)
4	27 (7.7%)	280 (80.0%)	49 (10.1%)	529 (77.1%)	25 (7.2%)	329 (83.1%)
Missing	0	1	0	0	34	9

### GWAS Comparing IDHmut Versus IDHwt Adult Diffuse Glioma

**Figure 2.**
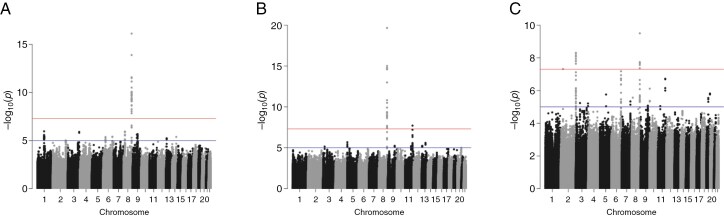
Manhattan plots denoting results for comparing *IDH*mut versus *IDH*wt glioma in the (a) Mayo Affymetrix GWAS, (b) Mayo/UCSF Illumina GWAS, and (c) TCGA GWAS. Red horizontal line denotes *P* = 5 × 10^−8^ and blue line denotes *P* = 5 × 10^−6^.

A case–case GWAS was performed in the three independent datasets to identify variants that have a different effect in *IDH*mut patients versus *IDH*wt (**Figure 2**). Seventy variants passed genomewide significance (*P* < 5 × 10^−8^) in the corresponding meta-analysis (**Supplementary Table S1**). Of the 70 variants, 34 were in the *CCDC26* region, with the most significant being rs55705857 (meta *P* = 1.35 × 10^−43^, Mayo GWAS OR = 5.13; **Table 3**). Notably, rs55705857 was previously demonstrated to be a causal risk variant for *IDH*mut glioma.^1^ The remaining variants were also in two known glioma regions. Thirteen variants were in *PHLDB1* (most significant, rs7125115, meta *P* = 3.46 × 10^−17^, Mayo GWAS OR = 1.54). The *PHLDB1* germline variant, rs7125115, was previously observed to be a candidate functional variant for the *PHLDB1* region.^15^ Twenty-three variants were in *D2HGDH* (most significant, rs71430382, meta *P* = 2.43 × 10^−12^, Mayo GWAS OR = 1.55). The *D2HGDH* variant, rs71430382, was originally identified to be associated with risk of developing an *IDH*mut glioma via a case–control GWAS.^10^ Variants in *TERT* and *MAML2* were nominally significant in the meta-analysis; both regions were previously shown to be associated with risk of *IDH*wt and *IDH*mut glioma, respectively (**Supplementary Table S1**).

**Table 3.** Variants that Reached Genomewide Significance (*P* < 5 × 10^-8^) in the Meta-analysis for the Corresponding Case–Case Analysis. The odds ratio (OR) denotes the ratio of the relative risk for developing an *IDH*mut glioma versus the relative risk for developing an *IDH*wt glioma.

Chromosome	Gene	Variant	Previously Published Association	Allele	Mayo GWAS OR (95% CI)	Meta *P*-value	Meta *I*^2^
*IDHmut versus IDHwt*							
8	*CCDC26*	rs55705857	*IDH*mut	G	5.13 (3.49, 7.54)	1.35 × 10^-43^	34.9
11	*PHLDB1*	rs7125115	*IDH*mut	G^*^	1.54 (1.22, 1.93)	3.46 × 10^-17^	0
2	*D2HGDH*	rs71430382	*IDH*mut	T	1.55 (1.24, 1.94)	2.43 × 10^-12^	66
*IDHmut non-codeleted versus IDHwt*							
8	*CCDC26*	rs55705857	*IDH*mut	G^*^	4.56 (2.93, 7.08)	1.9 × 10^-26^	43
11	*PHLDB1*	rs7125115	*IDH*mut	G	1.87 (1.37, 2.56)	3.56 × 10^-14^	0
*IDHmut 1p/19-codeleted versus IDHwt*							
8	*CCDC26*	rs55705857	*IDH*mut	G^*^	5.74 (3.67, 8.97)	2.12 × 10^-33^	61.7
9	*CDKN2A/B*	rs1360589	*IDH*wt	C	0.77 (0.59, 1.01)	2.60 × 10^-8^	0
*Amongst subjects with AA genotype for rs55705857: IDHmut versus IDHwt*							
11	*PHLDB1*	rs7125115	*IDH*mut	G^*^	1.48 (1.11, 1.96)	1.73 × 10^-13^	0
2	*D2HGDH*	rs71430382	*IDH*mut	T	1.65 (1.25, 2.17)	8.86 × 10^-10^	72.7

^*^The allele and corresponding OR denotes the risk allele from the previously published association. The OR in the supplementary table corresponds to the complementary allele.

Analyses were also performed comparing the two primary *IDH*mut subtypes separately, *IDH*mut 1p/19q-codeleted versus *IDH*wt and *IDH*mut non-codeleted versus *IDH*wt (**Supplementary Figure S1**). When comparing *IDH*mut non-codeleted versus *IDH*wt, the *CCDC26* and *PHLDB1* regions were both genomewide significant with the corresponding most significant variants being rs55705857 and rs7125115, respectively (**Table 3**; **Supplementary Table S2**). Variants in *D2HGDH* were nominally significant in the meta-analysis. When comparing *IDH*mut 1p/19q-codeleted versus *IDH*wt, the *CCDC26* and *CDKN2A*/B regions were genomewide significant, with the corresponding most significant variants being rs55705857 and rs1360589, respectively (**Supplementary Table S2**). Variants in *D2HGDH* were nominally significant in the meta-analysis.

### GWAS Comparing IDHmut Versus IDHwt: Stratified by CCDC26 Variant rs55705857

The *CCDC26* variant rs55705857 was previously shown to be a causal risk variant for *IDH*mut glioma.^1^ However, it is important to understand the predisposition of developing an *IDH*mut glioma amongst patients that do not have the risk allele for rs55705857. Thus, a case–case GWAS was performed comparing *IDH*mut versus *IDH*wt among the subset of patients that do not carry the causal rs55705857 risk variant (**Supplementary Figure S2**). Among the subset of glioma patients that do not carry a risk allele for rs55705857 (ie, among patients that have genotype AA for rs55705857), 21 variants passed genomewide significance in the corresponding meta-analysis (**Table 3**; **Supplementary Table S3**). All variants were in two known glioma regions: 11 variants in *PHLDB1* (most significant rs7125115, meta *P* = 1.73 × 10^−13^, Mayo GWAS OR = 1.48) and 10 variants in *D2HGDH* (most significant rs71430382, meta *P* = 8.86 × 10^−10^, Mayo GWAS OR = 1.65). Variants in *MAML2* were nominally significant in the meta-analysis.

**Figure 3.**
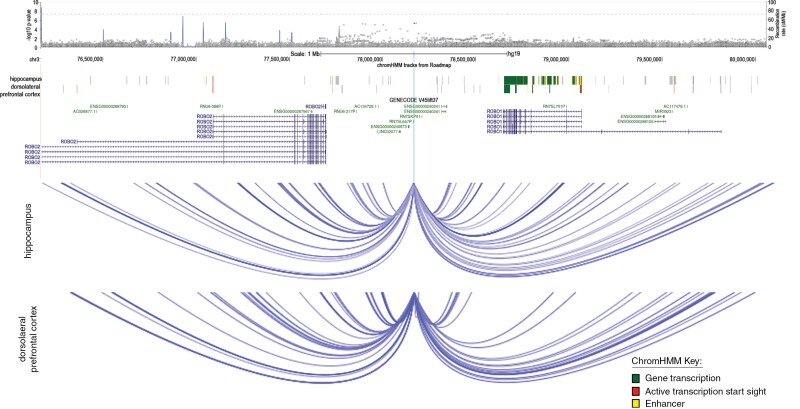
Locus zoom, Chrom HMM, and Hi-C results for the *ROBO* gene region. *P*-values in the locus zoom plot denote results from the Mayo Affymetrix case–case GWAS, the red dot denotes the most significant variant rs4680975, and the blue line shows recombination rates.

A GWAS was also performed among the subset of patients who had one or two risk alleles for rs55705857 (ie, AG or GG genotype) and zero variants were genomewide significant (**Supplementary Table S4**). Nominal significance was observed for four variants on chromosome arm 3p, located between the *ROBO1* and *ROBO2* genes (most significant, rs4680975, meta *P* = 4.65 × 10^−7^, Mayo GWAS OR = 0.11). These results suggest that among the subset of glioma patients that had at least one G-allele (the risk allele) for rs55705857, having a C-allele for rs4680975 confers a relative risk of an *IDH*wt glioma approximately nine times higher than the relative risk of developing an *IDH*mut glioma. No significant associations were observed between rs4680975 genotype and gene expression of *ROBO1* or *ROBO2* by eQTL in any normal brain tissue in the GTEx portal (data not shown). TCGA LGG and GBM datasets were evaluated separately to determine if rs4680975 genotype was associated with tumor expression of *ROBO1* or *ROBO2* (**Supplementary Figure S3**). There was a significant association with *ROBO1* expression among the subset of *IDH*wt LGG TCGA patients that had at least one risk allele for rs55705857 (*P* = .034); no association was observed with *ROBO2* expression (*P* = .73). As a negative control, similar analyses were performed for *IDH*mut LGG patients in TCGA and there was no significant association between rs4680975 genotype and either *ROBO1* or *ROBO2* gene expression among *IDH*mut patients that had at least one risk allele for rs55705857 (*P* = .80 and *P* = .36, respectively). Thus, a significant eQTL relationship was only observed for *IDH*wt patients that had the risk allele for rs55705857, which aligns with the observed GWAS results. In the GBM TCGA data, zero *IDH*wt patients carried both the rs55705857 and rs4680975 risk alleles; thus, an eQTL could not be performed. DNA–DNA interactions in normal adult hippocampus and dorsolateral prefrontal cortex support the potential interaction of the locus and the area surrounding the start site of *ROBO1* (**Figure 3**). This supports the hypothesis that the variant may influence expression of *ROBO1* in the brain through interactions with the promoter regions controlling expression.

### GWAS Comparing IDHmut Versus IDHwt: *Stratified by sex*

Glioma variants, including rs55705857, were shown to have sex-specific effects.^6,7^ Thus, a case–case GWAS was performed comparing *IDH*mut versus *IDH*wt, stratified by sex (**Supplementary Figure S4**). Among females, nine variants passed genomewide significance in the corresponding meta-analysis and all were in the *CCDC26* region, with the most significant variant being rs55705857 (meta *P* = 9.15 × 10^−24^, Mayo GWAS OR = 5.29) (**Supplementary Table S5**). Five variants in the *D2HGDH* region had nominal significance in the meta-analysis (most significant, rs71430382, meta *P* = 9.55 × 10^−7^, Mayo GWAS OR = 1.40). Among males, 29 variants passed genomewide significance in the meta-analysis comparing *IDH*mut versus *IDH*wt and all were in known glioma regions (**Supplementary Table S6**). Twenty-five of the variants were in the *CCDC26* region, with the most significant being rs55705857 (meta *P* = 5.95 × 10^−22^, Mayo GWAS OR = 5.01), and four variants were in *PHLDB1* (most significant rs7125115, meta *P* = 2.86 × 10^−12^, Mayo GWAS OR = 1.37). While the *CCDC26* variant rs55705857 has been shown to have a significantly larger OR in females versus males with respect to risk of developing glioma in case–control analyses,^6,7^ we did not observe a statistically significant different OR between females and males in the case–case analyses comparing *IDH*mut versus *IDH*wt glioma (**Supplementary Table S7**). Similarly, we did not observe statistically significant differences between females and males for the *D2HGDH* and *PHDLB1* variants in the case–case analyses.

### Development of Polygenic Risk Models to Predict IDH Tumor Mutation Status

Regression trees were developed to predict *IDH* tumor status. The models were developed using the 31 known germline variants from European and East Asian GWAS and age as candidate predictor variables: 27 variants reported in European GWAS, three variants reported in East-Asian GWAS, and the *ROBO1* variant described above. When only variants were considered, the final pruned model retained only rs55705857 for predicting *IDH* status (**Supplementary Figure S5a**). When age at diagnosis was also considered, then the final pruned model retained only age (**Supplementary Figure S5b**). Thus, while regression tree models evaluate interactions during the model building process, no interactions were retained in the final pruned models. Since interactions were not observed in the tree models, multivariable logistic regression with elastic net regularization was also used to develop models to predict *IDH* status. This resulted in a model that contained rs55705857, the *D2HGDH* variant rs71430382, and age (**Supplementary Table S8**). The validation AUC was 0.84 (95% CI: 0.82, 0.87) and 0.88 (95% CI: 0.85, 0.90) in the Mayo/UCSF and TCGA data, respectively. The AUCs for this model were similar for males and females, as shown by stratified AUC analysis (**Supplementary Figure S6**).

A subset of the patients in the Mayo Affymetrix and Mayo/UCSF cohorts had MRIs available to determine presence of contrast enhancement (**Supplementary Table S9**). In the Mayo Affymetrix cohort, 89% (117/132) of *IDH*wt and 48% (70/146) of *IDH*mut patients showed contrast enhancement on MRI. In the Mayo/UCSF cohort, 90% (125/139) of *IDH*wt and 62% (36/58) of *IDH*mut patients showed contrast enhancement on MRI. Logistic regression was used to determine if contrast enhancement improved the predictive performance of the model. As a sensitivity analysis and to evaluate performance on the smaller subset of patients that had MRI data available, we re-developed a model using the Mayo Affymetrix cohort that had MRI data. The validation AUC of the model containing rs55705857, rs71430382, and age was 0.84 (95% CI: 0.78, 0.90) on the Mayo/UCSF data, which aligned with the results above from the full cohort. When contrast enhancement was added as a predictor variable to the logistic model, the validation AUC on the Mayo/UCSF cohort was 0.84 (95% CI: 0.78, 0.91) (**Supplementary Table S10**).

The predictive ability of a polygenic risk model is typically evaluated using AUC. An AUC provides a measure of a test’s performance, that is, it evaluates sensitivity and specificity across all possible risk score thresholds. For clinical implementation of a polygenic risk model, a threshold needs to be chosen to classify a patient as *IDH*mut or *IDH*wt. As proof of concept, two different model score thresholds were evaluated from the logistic model that was developed on the full Mayo Affymetrix cohort, which contained rs55705857, rs71430382, and age as predictor variables and had validation AUCs of 0.84 and 0.88 (**Supplementary Table S8**). The first threshold was chosen assuming that the clinical goal was to maximize specificity, that is, to correctly classify *IDH*wt patients. To obtain 90% specificity in the Mayo Affymetrix data (where the model was developed) a score threshold of 0.4339 was necessary (**Supplementary Table S11**). Applying the corresponding threshold to the Mayo/UCSF and TCGA data resulted in model sensitivity of 68% and 65%, and specificity of 86% and 91%, respectively. The second threshold was chosen assuming that the clinical goal was to maximize sensitivity, that is, to correctly classify *IDH*mut patients. To obtain 90% sensitivity in the Mayo Affymetrix data, a score threshold of −0.7199 was necessary (**Supplementary Table S11**). Applying the corresponding threshold to the Mayo/UCSF and TCGA data resulted in sensitivity of 87% and 89%, and specificity of 58% and 68%, respectively.

## Discussion

We aimed to directly compare *IDH*mut and *IDH*wt glioma to understand the interaction between *IDH* tumor mutation and germline variants. While previous GWAS analyzed the risk of specific subtypes of adult glioma using a case–control design and identified germline variants associated with risk of specific subtypes (eg, risk of *IDH*mut or risk of *IDH*wt glioma), the analysis did not specifically test if the OR was different between subtypes. This nuance is important for prioritizing and designing follow-up functional experiments. We observed that there were statistically significant interactions between *IDH* tumor mutation status and *CCDC26* (rs55705857), *PHLDB1* (rs7125115), and *D2HGDH* (rs71430382) germline variants. While it is already known that the *CCDC26* is a causal variant for *IDH*mut glioma, these findings highlight the importance of prioritizing the *PHLDB1* and *D2HGDH* regions in future functional experiments. Interestingly, both regions also have tumor alterations. *D2HGDH* is located on chromosome region 2q37 and this region was shown to be frequently deleted in *IDH*mut non-codeleted glioma.^16^*PHLDB1* is located on 11q23.3, which is a region that is frequently gained in *IDH*mut non-codeleted glioma.^16^

Because ~60% of *IDH*mut glioma patients do not carry the rs55705857 causal variant, we also aimed to identify germline variants that are associated with *IDH* tumor mutation independent of rs55705857. By stratifying patients by their rs55705857 genotype, we were able to demonstrate that the *PHLDB1* and *D2HGDH* variants had significant interactions with *IDH* mutation status, independent of rs55705857. Specifically, among adult glioma patients that do not carry a risk allele (G-allele) for rs55705857, the *D2HGDH* and *PHLDB1* variants increased the likelihood of having a tumor that is *IDH*mut. A different variant in the *PHLDB1* region (rs498872) has been shown to be associated with risk of glioma in East Asians; however, the two variants are correlated.^14^ These findings demonstrate the need to evaluate the function and identify the causal variants for the *D2HGDH* and *PHLDB1* regions as these variants may explain why patients that do not have the rs55705857 causal variant develop *IDH*mut adult brain tumors. For example, we have previously shown that there is a strong eQTL between rs71430382 and *D2HGDH* (less expression of the mRNA with increased risk gene dosage).^10^ Since *D2HGDH* removes 2-hydroxyglutarate from within cells, a plausible mechanism of action may be that loss of *D2HGDH*, either by somatic deletion or by carriage of the risk allele, generates elevated or superelevated levels of the oncometabolite 2-hydroxyglurate.

A case–case GWAS was also performed within the subset of patients that carry at least one risk allele for rs55705857. This analysis identified novel variants between the roundabout guidance receptor 1 (*ROBO1)* and receptor 2 (*ROBO2*) genes that were associated with higher likelihood of being *IDH*wt (versus *IDH*mut) among glioma patients that carry the G-allele for rs55705857. That is, even though rs55705857 is a causal variant for development of *IDH*mut adult glioma, some patients who have a G-allele for rs55705857 will develop an *IDH*wt tumor. The eQTL analyses demonstrated a significant association between the most significant variant (rs4680975) in this region and tumor expression of *ROBO1*. Interestingly, statistical significance was only observed among *IDH*wt glioma that carry a G-allele for rs55705857, which directly aligns with the GWAS results. While the eQTL association aligns with the GWAS findings, the sample size for the eQTL analysis was small. Specifically, there were only six *IDH*wt LGG TCGA patients that carry the G-allele for rs55705857, of which two also carry the rs4680975 risk allele. Previous GWAS that analyzed GBM^17^ and IDHwt^10^ tumors did not identify this region; thus, there appears to be an interaction between *IDH* tumor mutation and rs55705857 germline genotype. The *ROBO* genes are promising therapeutic targets for cancer and are currently being evaluated as targets for GBM.^18^ In GBM development, the *ROBO* genes are involved through an association with *SLIT* genes in a ligand-receptor system that plays a role in cell migration, stem cell growth, angiogenesis, and tumor formation, and are implicated in myeloid related immunosuppression in the tumor microenvironment.^19,20^ The *ROBO*/*SLIT* interaction is associated with glioma grade and prognosis.^21^*ROBO1* was recently targeted by CAR-T cells in cell lines derived from a recurrent GBM xenograft model and mice receiving one dose of the CAR-T cells had a doubling in median survival in comparison to untreated mice.^22^ While the GWAS finding is very interesting, future experiments are necessary to understand the relationship of the *ROBO1/2* region and development of *IDH*wt tumors.

With respect to sex, it is known that males have a higher prevalence of glioma than females.^23^ Sex-specific germline risk associations have been reported for adult diffuse glioma^6,7^ and interactions between *IDH* tumor mutation and sex have been observed, particularly with respect to cellular composition.^24^ However, we did not observe statistically significant differences across males and females with respect to associations of germline variants and *IDH* tumor mutation status.

Polygenic models have been developed to predict *IDH* tumor mutation status for adult diffuse glioma,^8,9^ which would allow for prediction of molecular subtype prior to surgery and could inform appropriate individualized treatment. For example, molecular classification of tumors in eloquent areas of the brain that are not amenable to surgical efforts. Currently tissue is needed to determine molecular subtype; thus, the ability to predict molecular subtype using a non-invasive blood-based assay is clinically relevant, especially as the field begins to explore the utility of *IDH* inhibitor drugs. Published *IDH* mutation polygenic models were developed using only demographic information (age and sex), only germline variants, as well as integrating demographic and germline variants^8,9^ In a prior study, age, sex, and the full GWAS data (ie, > 1 million variants) were included in a simple additive regression model, which achieved a validation AUC of 0.895 (95% CI: 0.872–0.918) in TCGA data.^9^ In comparison, the multivariable logistic model discussed in our study utilized only two germline variants (rs55705857 and the *D2HGDH* variant rs71430382) and age and had a validation AUC of 0.88 (95% CI: 0.85, 0.90) in TCGA data. Including contrast enhancement on MRI as a predictor to the model did not improve the AUC. Previous publications have similarly reported polygenic risk models with high AUCs.^8,9^ Since validation AUCs have consistently been high, we took the next step and quantified sensitivity and specificity of models. To estimate sensitivity and specificity, a threshold needs to be chosen to convert the model predictions into two classes: a positive test (predict *IDH*mut) or a negative test (predict *IDH*wt). Choosing a threshold requires a tradeoff between whether the clinical goal is to maximize sensitivity (here, defined as correctly predicting *IDH*mut) or maximize specificity (correctly predicting *IDH*wt). Ultimately, the choice between maximizing sensitivity or specificity is dependent on the clinical application. We were unable to define thresholds that resulted in both high sensitivity and high specificity; however, this does not imply that polygenic based models will not be useful. Other techniques have been interrogated to develop models to predict *IDH* tumor status, or tumor grade, prior to surgery. This includes mass spectrometry,^25^ PET,^26,27^ and MRI-based machine learning models.^28–30^ All these models aimed to tailor surgical efforts or treatment to the tumor molecular subtype for improving personalized medicine. Ultimately, the optimal approach may combine the strengths of each technology, and future work should entail evaluating models that integrate these technologies.

Regardless of disease or cancer type, most GWAS studies have been performed on European populations.^31^ In glioma, the only non-European GWAS were conducted in East Asians, which identified three novel germline variants: in or near *STK38L*, *RAB27A*, and *CYP4F12*.^32,33^ Furthermore, only three of the variants identified in European GWAS were validated in East Asians (*TERT*, *PHLDB1*, and *RTEL1*).^14^ We compared the allele frequency of all 30 glioma germline variants identified previously and showed that there is large variation across populations, which likely explains why most adult glioma germline variants have not replicated across the European and East Asian GWAS. This discrepancy also demonstrates that polygenic risk scores are likely going to be population specific, and thus, there is significant need to perform GWAS studies in diverse populations. Further complicating matters, clinically relevant acquired brain tumor alterations have also been reported to vary across ancestries, suggesting that there is likely a genetic predisposition to additional acquired tumor alterations beyond *IDH* tumor mutation.^34–36^ For example, studies have shown that brain tumors in African Americans and Asians were more likely to have *TP53* mutations and *MDM2* amplification than Europeans, and less likely to have *EGFR* over-expression.^34,36^ In studies comparing East Asian and European ancestries it was observed that East Asians are also enriched for *H3F3A* positive and triple-negative glioma, whereas Europeans are enriched for *TERT* mutation and *CDKN2A/B* alterations.^4^ Thus, in addition to performing GWAS studies in more diverse populations, it is also critical to account for clinically relevant acquired tumor alterations in diverse populations.

Our study has limitations. Due to the limited sample size of non-Europeans in the available GWAS data, the analyses were only performed on patients of primary European ancestry. Due to the difference in allele frequency across genetic ancestries for the known glioma germline variants, it is critical that future GWAS studies be performed on diverse populations. Because of the limited sample size of datasets with molecular data, the discovery *P*-value threshold was relaxed to 1 × 10^−^4; however, the meta-analysis *P*-value was set at the genomewide level of 5 × 10^−8^. The validity of the results was further confirmed since most of the observed variants were known glioma variants. With the updated 2021 WHO diagnostic criteria, there has been effort to understand the differences between molecular GBM and classic (histologic) GBM.^37^ We were unable to differentiate these two groups of GBM across all three datasets, including differentiating undersampled GBM versus true molecular GBM.^37^ Similarly, we were also not able to compare *IDH1* versus *IDH2* mutations. With respect to developing polygenic risk models, none of the prior published approaches considered interactions between germline variants or between demographic/clinical variables and germline variants. We used regression trees that allow for interactions; however, the tree models did not detect significant interactions. An alternative, yet similar approach is random forest analysis, which essentially averages (ensembles) across hundreds or thousands of trees. The ensemble approach typically produces more accurate models, but at the cost of being highly complex and challenging to interpret. Lastly, it is interesting to note that while age at diagnosis was similar across the three datasets analyzed in this study, the *IDH*wt patients in the three datasets were younger than what is reported in CBTRUS (median = 65, IQR = [56,72]).^23^

Overall, we aimed to further understand the interaction between acquired *IDH* tumor mutation and germline variants to prioritize variants for future functional experiments. We further aimed to understand what germline variants are primarily responsible for development of *IDH*mut glioma beyond rs55705857, which is the only causal variant for *IDH*mut glioma identified to date. With respect to prioritizing future basic science experiments to identify additional causal variants, this work highlights that *D2HGDH* and *PHLDB1* should be prioritized for detailed functional studies. This study also demonstrated the importance of evaluating homogeneous glioma subgroups to identify novel germline variants that are associated with development of specific tumor alterations. For example, among glioma patients that carry the rs55705857 risk allele, novel variants near *ROBO1* were identified that increased the likelihood of having an *IDH*wt tumor (versus *IDH*mut tumor) by nine-fold. These results imply that even though rs55705857 is a causal variant for *IDH*mut glioma, if a glioma patient additionally has a C-allele for the *ROBO1* variant, then they are more likely to have an *IDH*wt versus an *IDH*mut tumor. Future experiments are necessary to validate the *ROBO1* variant, identify the causal variant, and define the functional mechanisms. Overall, development of tumor models that represent the genetic makeup of known causal variants will aid in more accurate models for interrogating therapeutics.

## Supplementary Material

vdaf147_suppl_Supplementary_Table_S1

vdaf147_suppl_Supplementary_Figures

vdaf147_suppl_Supplementary_Table_S2

vdaf147_suppl_Supplementary_Table_S3

vdaf147_suppl_Supplementary_Table_S4

vdaf147_suppl_Supplementary_Table_S5

vdaf147_suppl_Supplementary_Table_S6

vdaf147_suppl_Supplementary_Table_S7

vdaf147_suppl_Supplementary_Table_S8

vdaf147_suppl_Supplementary_Table_S9

vdaf147_suppl_Supplementary_Table_S10

vdaf147_suppl_Supplementary_Table_S11

## Data Availability

GWAS data are available at dbGaP Study Accession: phs003806.v1.p1
