# Supplementary material for: Dissecting the biology of gliomagenesis: Evaluating the interaction between IDH tumor mutation and germline variants
Source: Neurooncol Adv. 2025 Jul 8;7(1):vdaf147. doi: 10.1093/noajnl/vdaf147 (PMC12288032; doi:10.1093/noajnl/vdaf147)

SUPPLEMENTARY MATERIALS

Dissecting the biology of gliomagenesis: Evaluating the interaction between *IDH* tumor mutation and germline variants

Matthew L. Kosel, Paul A. Decker, Thomas M. Kollmeyer, Kristen L. Drucker, Anne K. Shurtz, Annette M. Molinaro, Gian Marco Conte, Mana Moassefi, Bradley J. Erickson, John K. Wiencke, Stephen Francis, Terry C. Burns, Rachel A. Vaubel, Margaret Wrensch, Daniel H. Lachance, W. Oliver Tobin, Robert B. Jenkins, Jeanette E. Eckel-Passow

SUPPLEMENTARY TABLES

Supplementary Table S1: Case-case GWAS comparing *IDH*mut versus *IDH*wt glioma.

Supplementary Table S2: Case-only GWAS using multinomial logistic regression comparing IDHmut codel, IDHmut noncodel, and IDHwt glioma; IDHwt was modeled as the reference group.

Supplementary Table S3: Case-case GWAS comparing *IDH*mut versus *IDH*wt, among patients with AA genotype for *CCDC26* variant rs55705857.

Supplementary Table S4: Case-case GWAS comparing *IDH*mut versus *IDH*wt, among patients with GG or AG genotype for *CCDC26* variant rs55705857.

Supplementary Table S5: Case-case GWAS comparing *IDH*mut versus *IDH*wt, among females.

Supplementary Table S6: Case-case GWAS comparing *IDH*mut versus *IDH*wt, among males.

Supplementary Table S7: Testing for differences in odds ratios between males and females for the variants that were significant in the sex-stratified GWAS analyses.

Supplementary Table S8: Coefficients from the multivariable logistic regression model.

Supplementary Table S9: Demographic information for the two GWAS datasets for the subset of patients that have data regarding presence of contrast enhancement on MRI.

Supplementary Table S10: Multivariable logistic model for predicting IDH tumor mutation status, from the subset of patients with MRI data.

Supplementary Table S11: Sensitivity and specificity from the multivariable logistic model described in Supplementary Table S8.

SUPPLEMENTARY FIGURES

Supplementary Figure S1: Manhattan plots representing the case-only GWAS results from the multinomial model comparing IDHmut codel versus IDHwt in (a) Mayo Affymetrix GWAS, (b) Mayo/UCSF Illumina GWAS, and (c) TCGA GWAS, and IDHmut noncodel versus IDHwt in (d) Mayo Affymetrix GWAS, (e) Mayo/UCSF Illumina GWAS, and (f) TCGA GWAS. The red horizontal lines denote $5x10^{-8}$ and the blue line denotes $5x10^{-6}$.

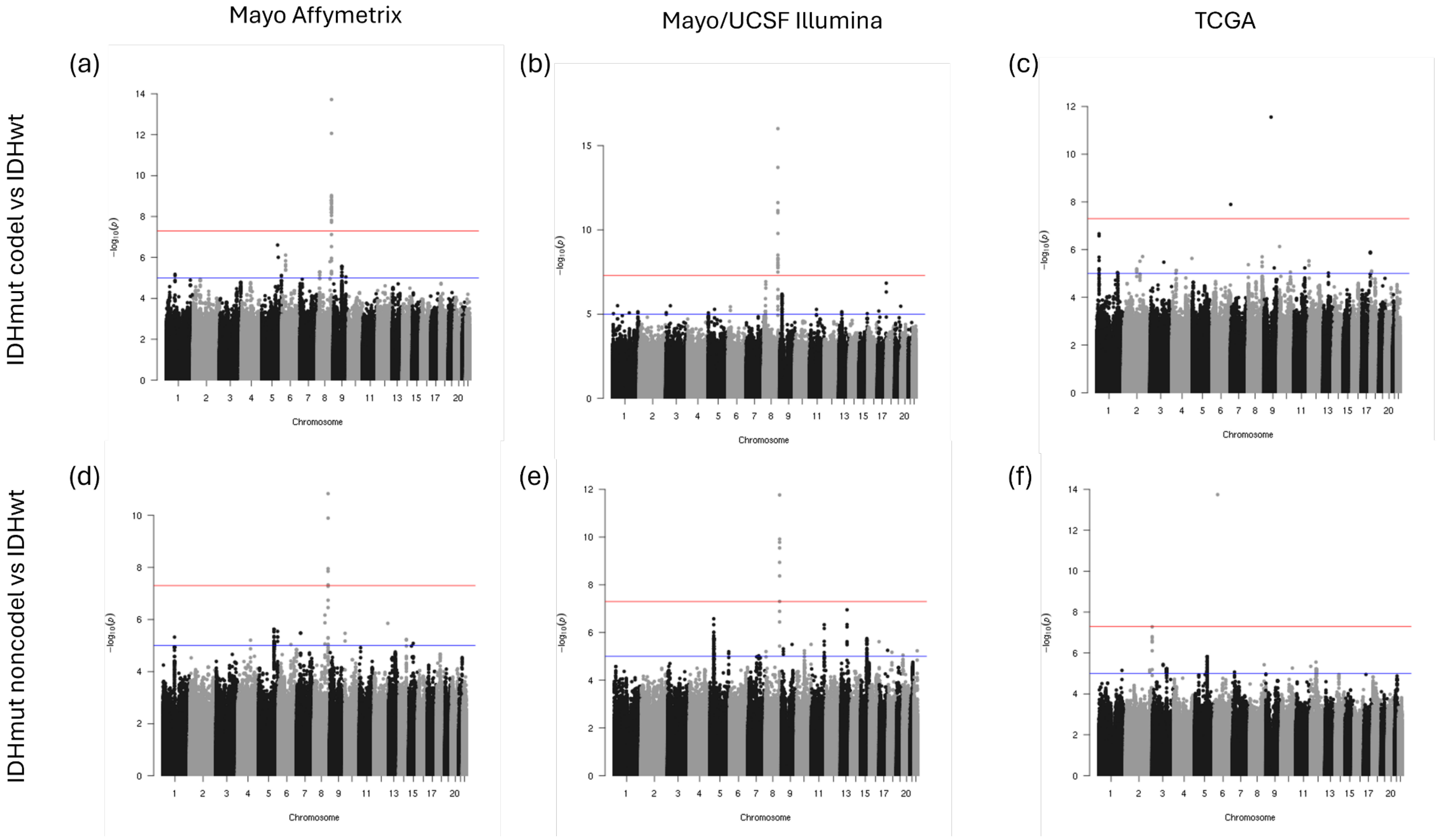

Supplementary Figure S2: Manhattan plots representing GWAS results for comparing *IDH*mut versus *IDH*wt, stratified by *CCDC26* variant rs55705857. Results for patients with AA genotype for rs55705857 in (a) Mayo Affymetrix GWAS, (b) Mayo/UCSF Illumina GWAS, and (c) TCGA GWAS, and GWAS results for patients with AG or GG genotype for rs55705857 in (d) Mayo Affymetrix GWAS, (e) Mayo/UCSF Illumina GWAS, and (f) TCGA GWAS. The red horizontal lines denote $5x10^{-8}$ and the blue line denotes $5x10^{-6}$.

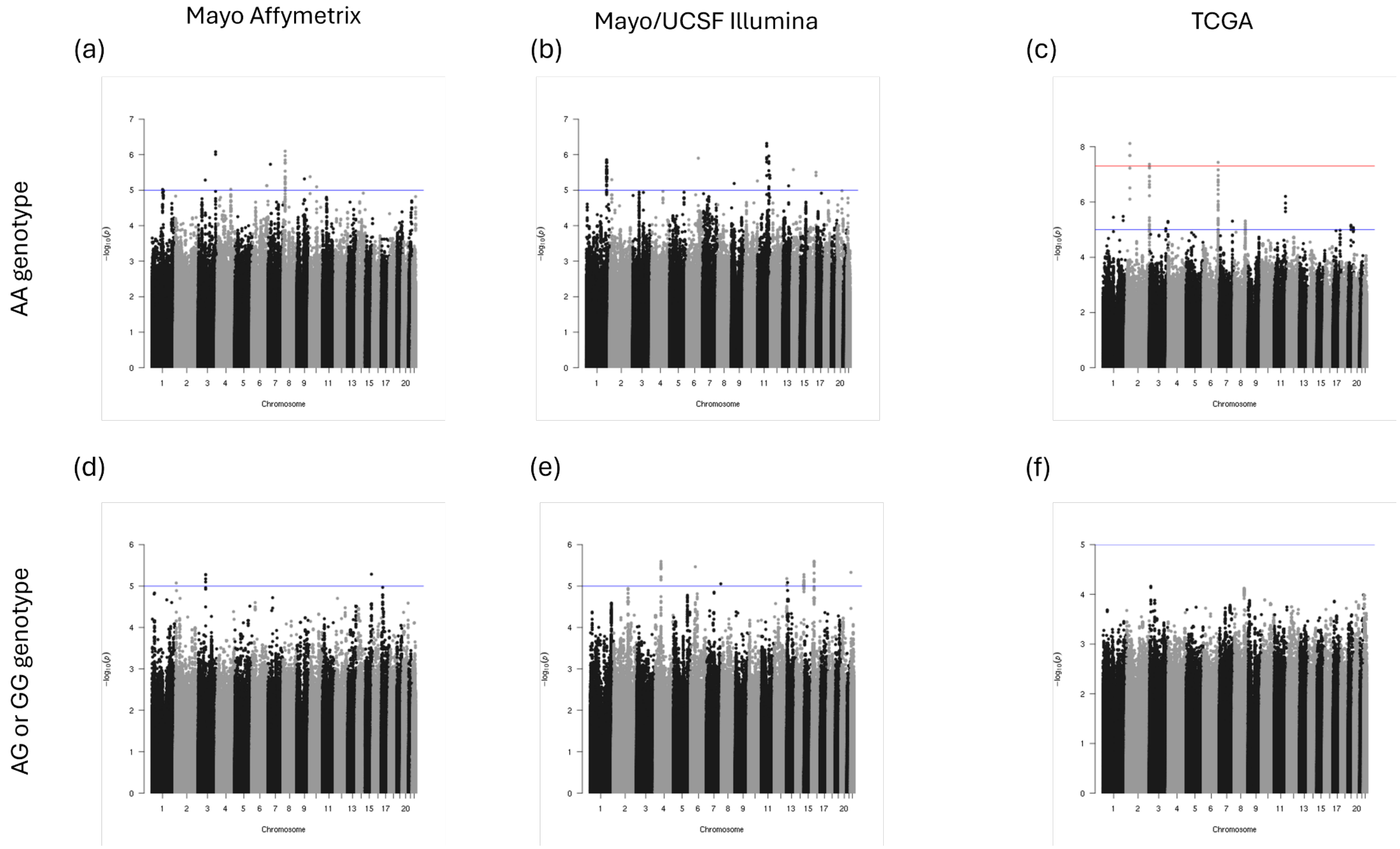

Supplementary Figure S3: TCGA LGG data were used to perform eQTL analyses between germline variant rs4680975 and *ROBO1* tumor gene expression in (a) all *IDH*wt tumors, (b) *IDH*wt tumors that have AG or GG genotype for *CCDC26* variant rs55705857, (c) all *IDH*mut tumors, and (d) *IDH*mut tumors that have AG or GG genotype for rs55705857. Similar eQTL analyses were performed between rs4680975 germline variant and *ROBO2* tumor gene expression in (e) all *IDH*wt tumors, (f) *IDH*wt tumors that have AG or GG genotype for *CCDC26* variant rs55705857, (g) all *IDH*mut tumors, and (h) *IDH*mut tumors that have AG or GG genotype for rs55705857. P-values derived from a linear regression model. The germline variants were imputed; hence, they contain values between zero and two. In the GBM TCGA data, zero *IDH*wt patients carried both the rs55705857 and rs4680975 risk alleles.

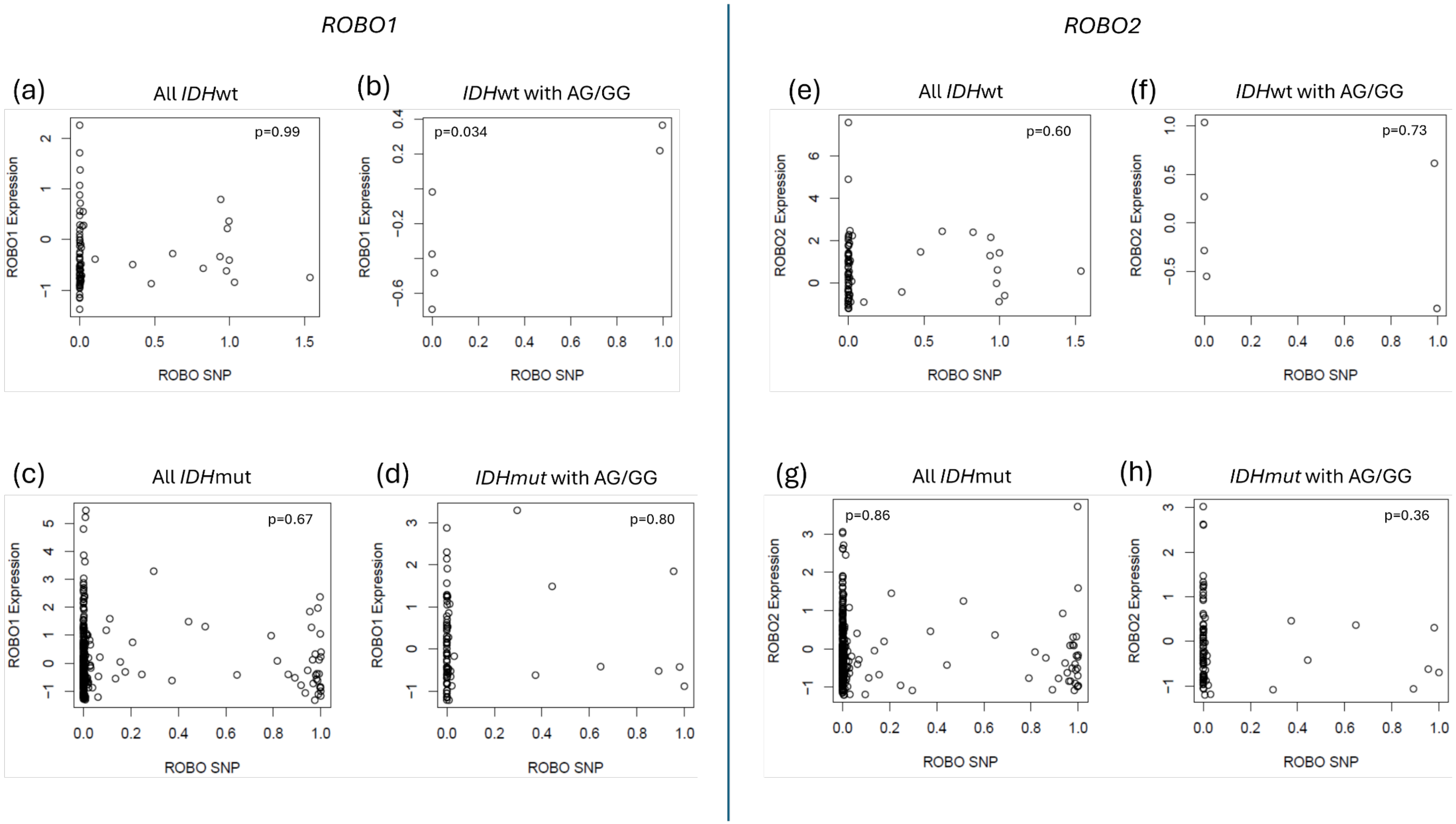

Supplementary Figure S4: Manhattan plots representing the GWAS results comparing *IDH*mut versus *IDH*wt, stratified by sex. Results for females in (a) Mayo Affymetrix GWAS, (b) Mayo/UCSF Illumina GWAS, and (c) TCGA GWAS, and results for males in (d) Mayo Affymetrix GWAS, (e) Mayo/UCSF Illumina GWAS, and (f) TCGA GWAS. The red horizontal lines denote $5x10^{-8}$ and the blue line denotes $5x10^{-6}$.

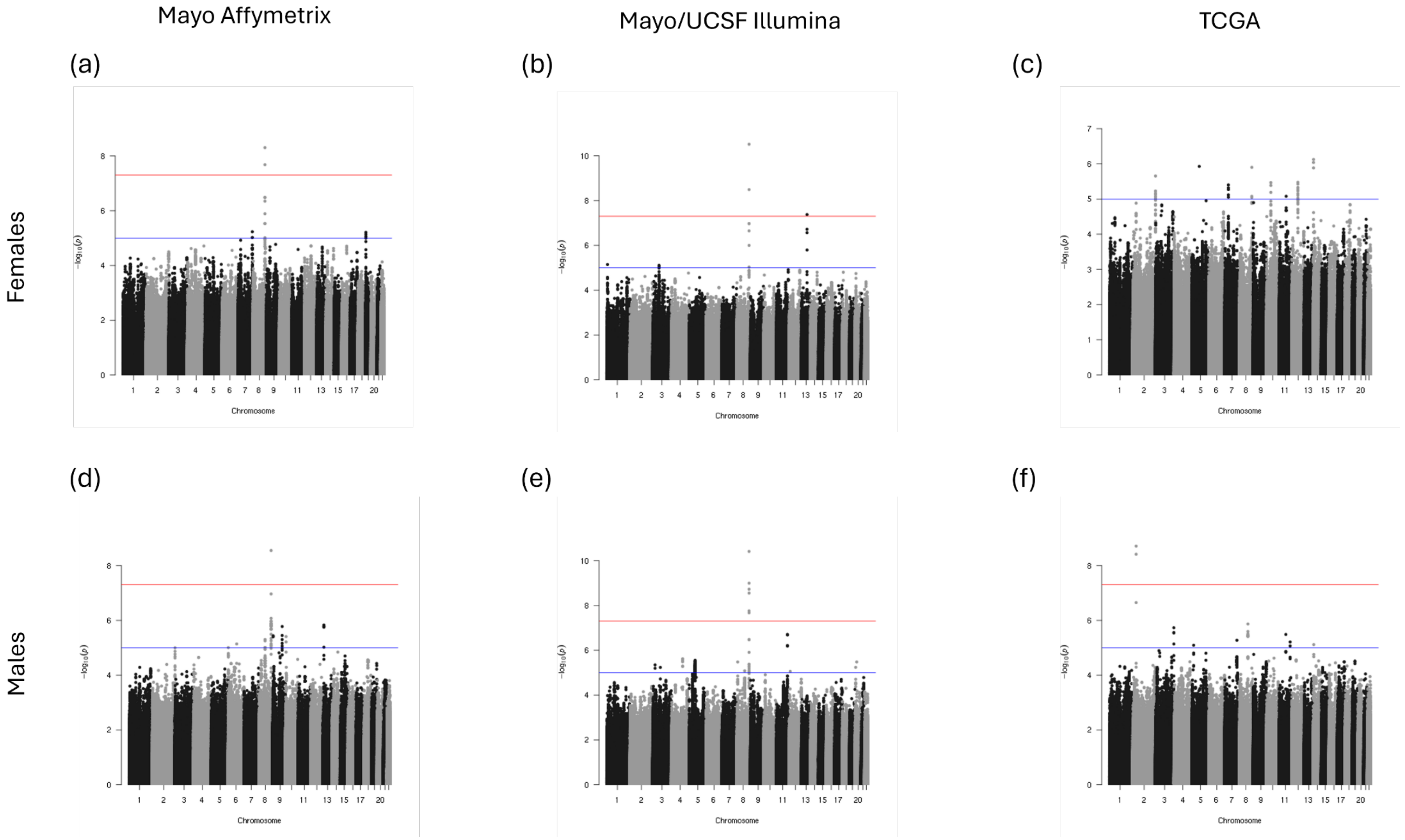

Supplementary Figure S5: (a) Regression tree model that was developed using only the 31 known glioma variants as candidate predictor variables, and the corresponding pruned tree. (b) Regression tree model that was developed using age at diagnosis and the 31 known glioma variants as candidate predictor variables, and the corresponding pruned tree. A blue box denotes prediction as IDHmut (MUT) and a green box denotes prediction as IDHwt (WT). The middle number in each box denotes the percent of IDHwt in the corresponding node; hence, a percentage ≥0.50 will be predicted as IDHwt and <0.50 predicted as IDHmut. The third number in each box denotes the percent of the total cohort that was used to develop the model that falls into the corresponding node. The tree models were developed using the Mayo Affymetrix data. If the variable is true (Yes), then you move to the left, and if it is not true (No), then you move to the right. For example, “rs55705857.A<1.5” denotes that if the genotype for germline variant rs55705857 has fewer than 1.5 copies of the A allele, then you move to the left; otherwise, to the right. Due to imputation, the variants are continuous variables and not necessarily integers.

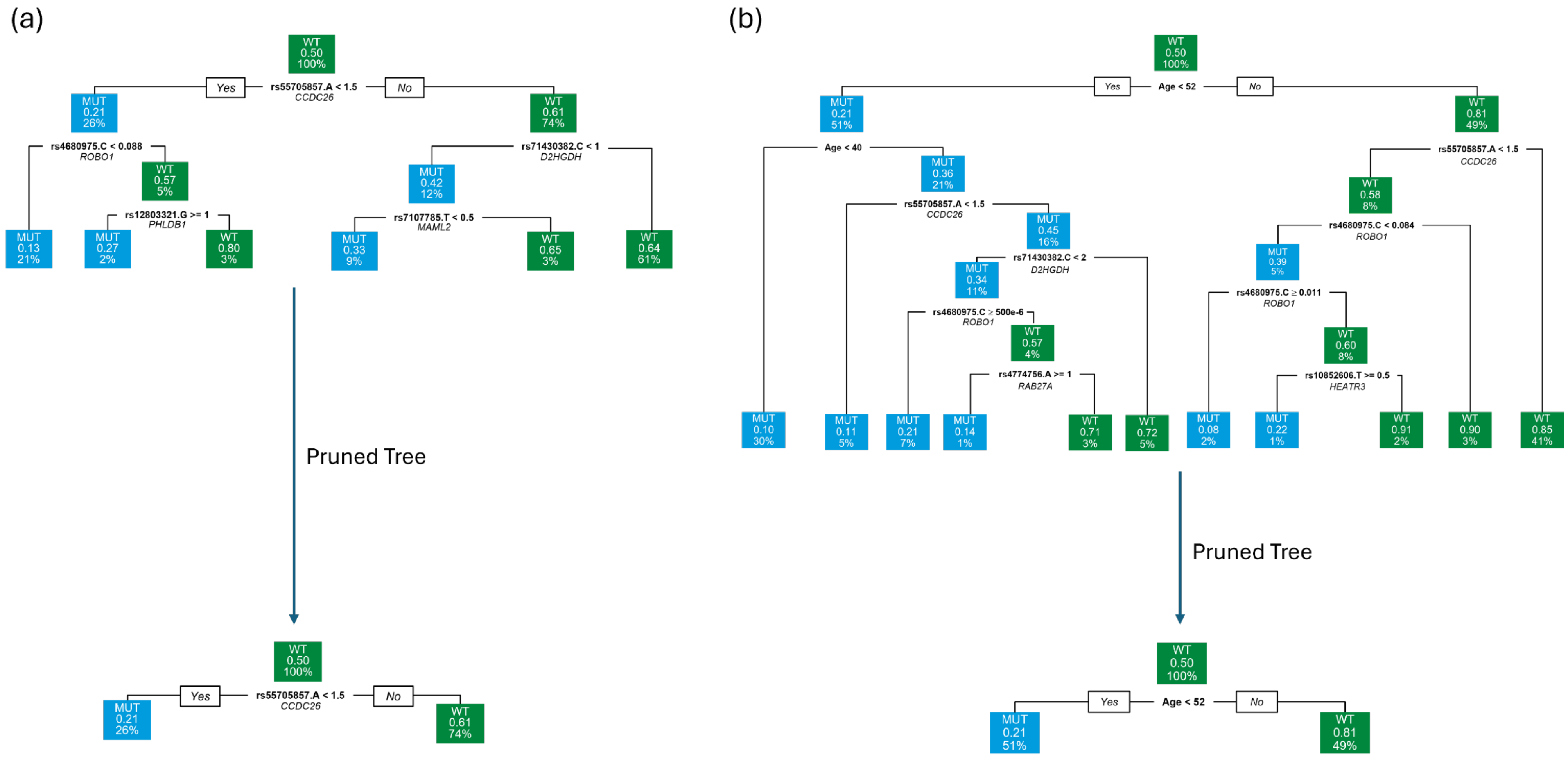

Supplementary Figure S6: Stratified AUC by sex for the multivariable logistic model applied to (a) Mayo/UCSF Illumina cohort and (b) TCGA.

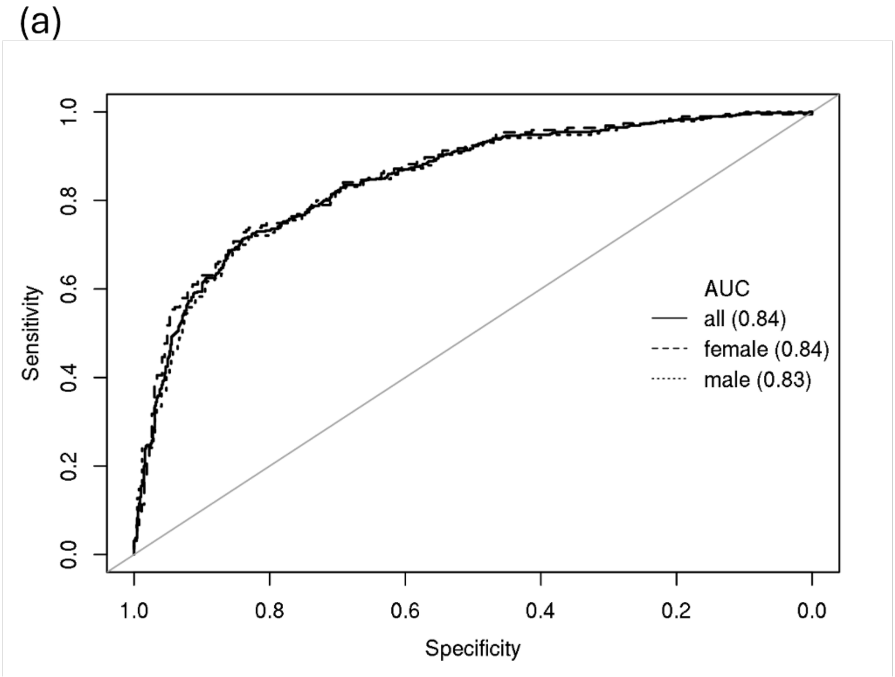

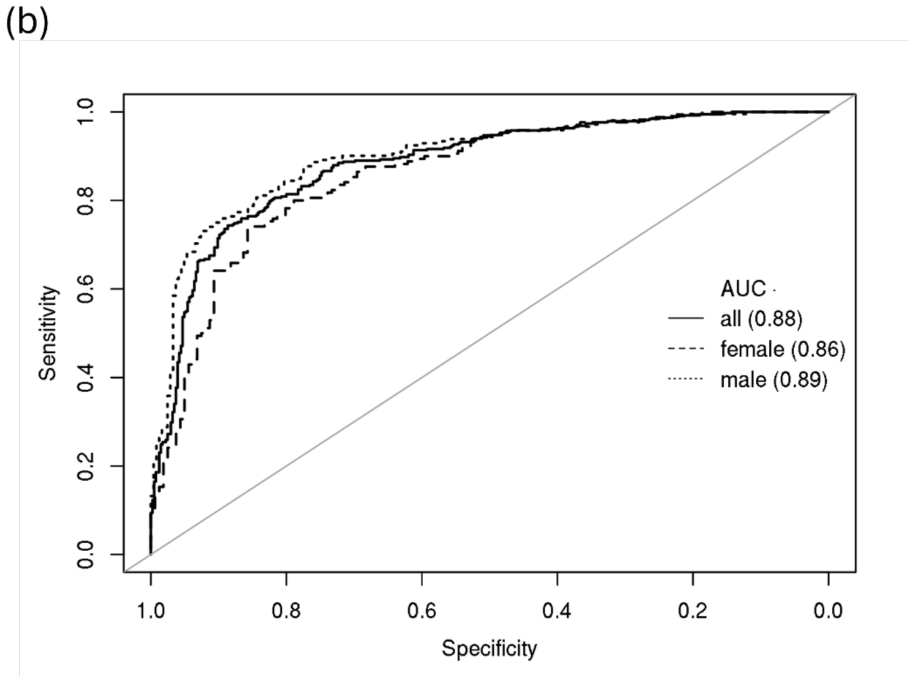

Supplement: vdaf147_suppl_Supplementary_Figures [file vdaf147_suppl_supplementary_figures.pdf]
